# Rab8 and TNPO1 function as the ciliary transport adapters for GPCRs

**DOI:** 10.1016/j.jbc.2026.112202

**Published:** 2026-04-24

**Authors:** Divyanshu Mahajan, Viswanadh Madugula, Hui Min Chia, Lei Lu

**Affiliations:** School of Biological Sciences, Nanyang Technological University, Singapore

**Keywords:** cilium targeting, cilium, GPCR, Rab8, TNPO1

## Abstract

The sensory functions of the primary cilium require the specific enrichment of a variety of transmembrane receptors, including G protein–coupled receptors (GPCRs). However, the molecular and cellular mechanisms governing the ciliary targeting of these receptors remain poorly understood. In our previous work, we identified Rab8 and TNPO1 as potential ciliary transport adapters for a range of ciliary membrane proteins, including PKHD1, RP2, RDH8, ARL13B, and RHO (a GPCR), through the formation of a ternary complex with their respective ciliary targeting sequences (CTSs). In this study, we screened nine cilium-localized GPCRs and identified five that interact with both Rab8 and TNPO1 and require the two adapters for ciliary localization. We found that β2 adrenergic receptor contains two CTSs: one in the third intracellular loop and another in the C-terminal cytosolic tail. Only the C-terminal CTS interacts with Rab8 and TNPO1, and its ciliary localization is dependent on both adapters. Our study suggests that Rab8 and TNPO1 might function as ciliary transport adapters for a significant number of GPCRs.

The primary cilium (hereafter cilium) is a hair-like cell surface protrusion that functions as an antenna, sensing and responding to the extracellular environment (1, 2, 3). Its sensory function depends critically on the specific enrichment of membrane proteins, such as G protein–coupled receptors (GPCRs), receptor tyrosine kinases, and ion channels. Membrane proteins are synthesized in the ER and follow the conventional secretory pathway to various cellular organelles. However, how ciliary membrane proteins are selectively targeted to the cilium instead of the plasma membrane (PM) remains puzzling, given that the ciliary membrane is a specialized PM domain sharing continuity with the PM. This selectivity is thought to be mediated by the ciliary diffusion barrier located at the transition zone near the ciliary base (4, 5, 6), although the detailed molecular and cellular mechanisms underlying this process remain unclear.

Cilium-enriched proteins typically possess ciliary targeting sequences (CTSs), short cytosolic amino acid motifs recognized by ciliary transport adapters. These adapters bind to and transport their clients across the ciliary diffusion barrier into the cilium interior (7, 8, 9, 10, 11). Unlike membrane trafficking in the secretory or endocytic pathways, ciliary transport does not involve membrane fission or fusion, relying instead on distinct mechanisms and machinery (9). Although CTSs lack a consensus sequence, two transport adapters, TULP3 and the Rab8-TNPO1 complex (hereafter, Rab8-TNPO1), have been reported. TULP3 has been implicated as the adapter for multiple GPCRs, the polycystin half complex, and PKHD1 (fibrocystin) by linking these clients to the IFT-A complex (12, 13, 14).

We propose that Rab8-GDP and TNPO1 together function as a ciliary transport adapter (9, 15, 16). Rab8 is essential for ciliary trafficking and ciliogenesis (17) and mediates vesicular delivery of several ciliary cargos, such as RHO (rhodopsin, a GPCR) (18), polycystin half complex (19, 20), and PKHD1 (21). TNPO1 (importin-β2/transportin-1) is a conserved cargo receptor for nucleocytoplasmic import (22, 23, 24), guiding cargo through the nuclear pore diffusion barrier, and releasing it upon binding to Ran-GTP inside the nucleoplasm. Beyond the nucleus, TNPO1 also mediates protein entry into the primary cilium (25, 26).

We previously found that Rab8-GDP, but not Rab8-GTP, cooperatively binds to the CTS with TNPO1, promoting ciliary localization (15). The ternary complex of Rab8-GDP, TNPO1, and the CTS facilitates the client's crossing of the ciliary diffusion barrier. Once inside the cilium, the complex disassembles through guanine nucleotide exchange of Rab8 from GDP to GTP, mediated by cilium-enriched exchange factors, enabling client release. This model explains how cargos with high diffusion mobility can be enriched within the cilium without requiring anchorage (9, 15). So far, we have identified five clients of Rab8-TNPO1: ARL13B, PKHD1, RDH8 (prRDH), RHO, and RP2 (15, 16). We expect further clients to be uncovered.

GPCRs, which comprise ∼1000 members in the human proteome (27), are among the most clinically and pharmacologically relevant receptors. Many GPCRs function in cilia, but the targeting mechanisms of these receptors remain unclear. Previous studies suggested TULP3 as a ciliary transport adapter for some GPCRs, based on reduced ciliary localization upon TULP3 depletion or KO (12, 13, 14, 28, 29, 30). In this study, we screened nine cilium-localized GPCRs and identified five that interact with TNPO1 in a Rab8-GDP-dependent manner. The ciliary localization of these GPCRs decreased upon the knockdown of Rab8 or TNPO1. Using β2 adrenergic receptor (ADRB2) as an example, we demonstrated that Rab8-TNPO1 binds to its CTS at the C-terminal cytosolic tail to facilitate its ciliary localization. Our findings reveal Rab8-TNPO1 as a novel ciliary transport adapter for GPCRs, thereby expanding the list of its clients.

## Results

### Cloning cilium-localized GPCRs

**Figure 1:**
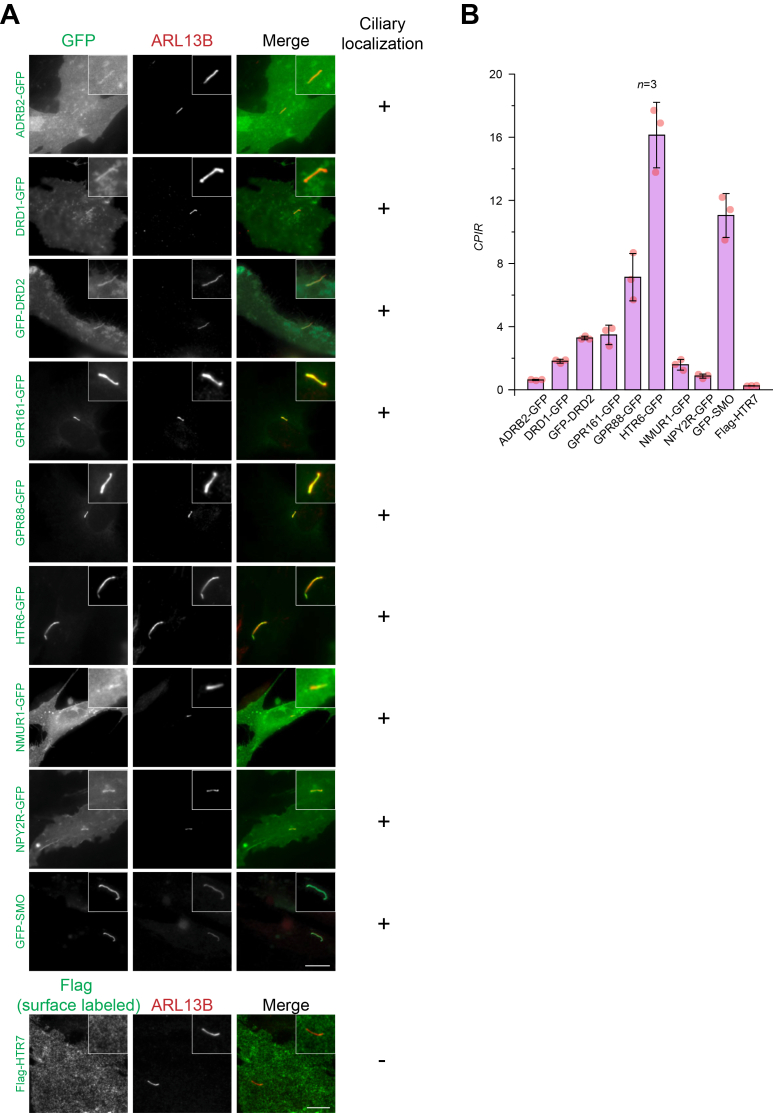
**The GPCRs used in this study localize to the cilium**. *A*, RPE1 cells transiently expressing indicated GFP- or Flag-tagged GPCR were fixed and processed for immunofluorescence. Cilia were identified by immunostaining endogenous ARL13B. In each image, the cilium of interest is enlarged and highlighted in a box at the upper right corner. Flag-HTR7 served as a nonciliary GPCR (negative control) and was detected by surface staining with anti-Flag antibody. The scale bar represents 10 μm. *B*, *CPIR* values of GPCRs in (*A*). *n* indicates the number of independent experiments each with ≥ 30 cells analyzed; error bars represent mean ± *SD*. *CPIR*, cilium-to-plasma membrane intensity ratio; GPCR, G protein–coupled receptor; HTR7, 5-hydroxytryptamine receptor 7.

Some of the rhodopsin-like (class A) and Smoothened/Frizzled-like (class F) GPCRs are known to localize and function at the cilium (31, 32, 33). To investigate the molecular mechanisms underlying their ciliary targeting, we acquired or cloned eight class A GPCRs (Table 1): ADRB2, dopamine receptor D1 (DRD1), dopamine receptor D2 (DRD2), G protein–coupled receptor 161 (GPR161), G protein–coupled receptor 88 (GPR88), 5-hydroxytryptamine receptor 6 (HTR6), neuromedin U receptor 1 (NMUR1), and neuropeptide Y receptor Y2 (NPY2R). In addition, we cloned one class F GPCR, SMO (smoothened) (Table 1). To facilitate imaging and detection, each GPCR was fused with the GFP tag. These constructs were transiently transfected into hTERT-RPE1 (hereafter RPE1) cells, and ciliogenesis was induced by serum starvation. The ciliary localization of these GPCRs was confirmed by costaining with the endogenous ciliary marker ARL13B (Fig. 1*A*). As a negative control, we selected Flag-tagged 5-hydroxytryptamine receptor 7 (HTR7), previously reported to be nonciliary (28). Flag-HTR7 was transiently expressed in RPE1 cells and detected by surface staining.

**Table 1:** Summary of ten GPCRs: their interaction with Rab8-TNPO1 and ciliary localization upon the knockdown of Rab8-TNPO1 or TULP3

GPCR	Cilium localization in RPE1	Rab8-TNPO1 interaction	Cilium localization reduction upon comptonization of:	
			Rab8-TNPO1	TULP3
ADRB2	+	+(CTT)	+	-a
DRD1	+	-	-	+^a^
DRD2	+	+	+	+^a^
GPR161	+	-	-	+^b^
GPR88	+	+	+	+^a^
HTR6	+	-	-	+^c^
NMUR1	+	+	+	ND
NPY2R	+	+	+	+^d^
RHO	ND	+^e^	ND	ND
SMO	+	-	-	ND

The data are from multiple sources, as indicated by respective superscript.

“+” indicates the positive interaction between Rab8-TNPO1 and GPCR or the compromised ciliary localization upon the knockdown of the indicated protein.

“-“ indicates the negative interaction between Rab8-TNPO1 and GPCR or the KD of indicated protein does not affect the ciliary localization of GPCR.

ND, not determined.

a Badgandi et al., 2017 (12).

b Mukhopadhyay et al., 2013 (14).

c Barbeito et al., 2021 (28).

d Loktev and Jackson, 2013 (30).

e Madugula et al., 2016 (15).

The cilium-to-plasma membrane intensity ratio (*CPIR*) provides a robust quantitative metric for ciliary localization, independent of the expression level of the protein of interest (15). Although the surface-labeling method offers better measurements of the PM and ciliary intensity, it requires extracellular labeling of the ciliary protein and is therefore applicable only to Flag-HTR7. However, as the GFP tags on our GPCRs are cytosolically exposed, this method could not be applied in this study. Consequently, the measured PM intensity might include intracellular GPCR pools, such as those in the ER or endosomes. Therefore, in this study, the *CPIR* primarily reflects the relative enrichment of a GPCR in the cilium compared to its presence in other subcellular pools (Fig. 1*B*). Consistent with the previous report (28), Flag-HTR7 exhibited no cilium enrichment, as indicated by its low *CPIR* value (Fig. 1*B*). The observed ciliary localization of these GPCRs aligns with previous reports (3), supporting the functionality of our constructs.

### Rab8-TNPO1 synergistically interact with cilium-localized GPCRs

We previously demonstrated that Rab8-TNPO1 function as a ciliary transport adapter for three ciliary transmembrane proteins (PKHD1, RDH8, and RHO) and two peripheral membrane proteins (ARL13B and RP2) (15, 16). To explore whether Rab8-TNPO1 functions as a ciliary transport adapter for ciliary GPCRs, we tested their interactions using pull-down assays. Specifically, we employed two complementary methods, utilizing either Rab8 or TNPO1 as bead-immobilized baits.

**Figure 2:**
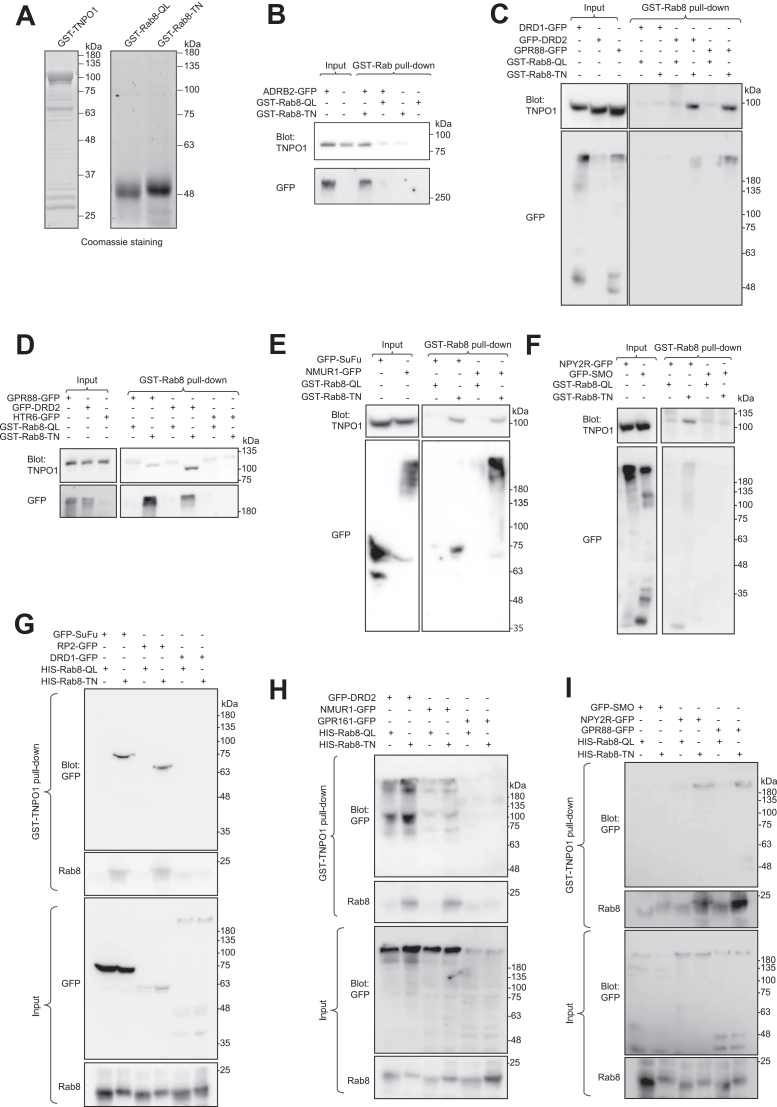
**Rab8-TN and TNPO1 form a ternary complex with certain GPCRs.** All cell lysates were from HEK293T cells. *A*, Coomassie staining of purified GST-tagged fusion proteins used for pull-down experiments. *B*, *C*, *D*, *E*, and *F*, GST-Rab8-TN specifically pulls down GPCRs and TNPO1. Bead-immobilized GST-Rab8-QL and -TN were incubated with lysates from cells transiently expressing GFP-tagged GPCRs. Retained proteins were analyzed by immunoblotting for GFP and endogenous TNPO1. The number of independent replicates that yielded similar results: ADRB2-GFP (*n* = 3), DRD1-GFP (*n* = 6), GFP-DRD2 (*n* = 2), GPR161-GFP (*n* = 2), GPR88-GFP (*n* = 3), HTR6-GFP (*n* = 2), NMUR1-GFP (*n* = 2), NPY2R-GFP (*n* = 2), and GFP-SMO (*n* = 4). *G*, *H*, and *I*, GST-TNPO1 specifically pulls down GPCRs in a Rab8-TN-dependent manner. Bead-immobilized GST-TNPO1 was incubated with lysates from cells transiently expressing GFP-tagged GPCRs, along with either purified His-tagged Rab8-QL or -TN. Retained proteins were immunoblotted for GFP and Rab8. The number of independent replicates that yielded similar results: DRD1-GFP (*n* = 3), GFP-DRD2 (*n* = 2), GPR161-GFP (*n* = 2), GPR88-GFP (*n* = 3), HTR6-GFP (*n* = 2), NMUR1-GFP (*n* = 2), NPY2R-GFP (*n* = 2), and GFP-SMO (*n* = 2). Molecular weight markers (kDa) are labeled to the right of all immunoblots. GPCR, G protein–coupled receptor; ADRB2, β2 adrenergic receptor; DRD1, dopamine receptor D1; DRD2, dopamine receptor D2; GPR161; G protein-coupled receptor 161; GPR88, G protein-coupled receptor 88; HTR6, 5-hydroxytryptamine receptor 6; NMUR1, neuromedin U receptor 1; NPY2R, neuropeptide Y receptor Y2; SMO, smoothened.

In the first method with Rab8 as the pull-down bait, we purified recombinant GST-tagged Rab8 proteins with GDP- or GTP-bound point mutations (T27N and Q67L, referred to as TN and QL, respectively) (34) (Fig. 2*A*). Bead-immobilized GST-Rab8-TN or QL was incubated with HEK293T cell lysates transiently expressing GFP-tagged ciliary GPCRs (Fig. 2, *B*–*F*). After extensive washing, bead-retained proteins, including GFP-tagged GPCRs and endogenous TNPO1, were detected by Western blotting. We observed that Rab8-TN, but not Rab8-QL, specifically pulled down exogenously expressed ADRB2, DRD2, GPR88, NPY2R, and NMUR1 (Fig. 2, *B*–*F*). In contrast, neither Rab8-TN nor Rab8-QL pulled down DRD1, HTR6, or SMO (Fig. 2, *C* and *D*, and *F*). Furthermore, Rab8-TN pulled down endogenous TNPO1 only when the GPCR interacted with Rab8-TN (Fig. 2, *B*–*F*), suggesting the formation of a Rab8-TNPO1-client ternary complex, similar to what we previously reported (15, 16).

In the alternative pull-down method using TNPO1 as the bait, we prepared recombinant GST-tagged TNPO1 and His-tagged Rab8-TN or Rab8-QL proteins. Bead-immobilized GST-TNPO1 was incubated with the same cell lysates expressing GFP-tagged ciliary GPCRs, along with His-tagged Rab8-TN or QL (Fig. 2, *G*–*I*). Consistent with the first method, GST-TNPO1 pulled down RP2 (positive control) (15), DRD2, GPR88, NPY2R, and NMUR1, but only in the presence of His-tagged Rab8-TN (Fig. 2, *G*–*I*). No interactions were observed with DRD1, GPR161, or SMO, regardless of whether Rab8-TN or QL was added (Fig. 2, *G*–*I*).

Interestingly, in both pull-down methods, Rab8-TN and TNPO1 also selectively pulled down exogenously expressed SuFu (Fig. 2, *E* and *G*), a cilium-localized soluble protein involved in hedgehog signaling. This observation suggests that Rab8-TNPO1 might serve as a ciliary transport adapter for SuFu. However, the functional significance of this interaction remains to be investigated.

### Rab8-TNPO1 is essential for the ciliary enrichment of GPCRs

**Figure 3:**
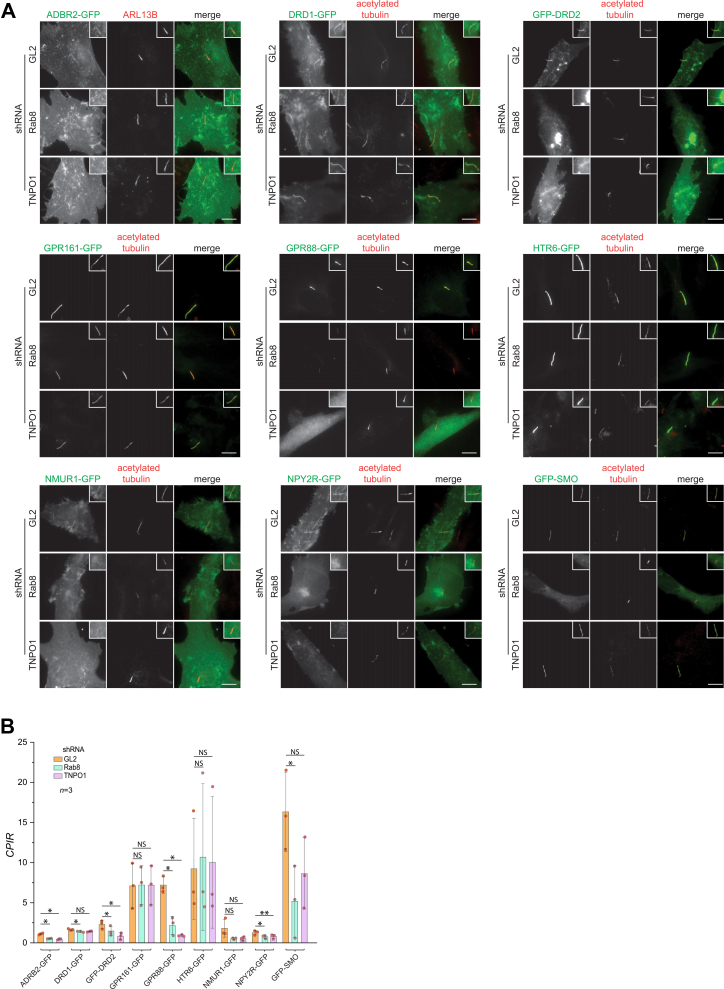
**Depletion of cellular Rab8 or TNPO1 decreases the ciliary localization of GPCRs**. Rab8 and TNPO1 were knocked down by the relevant shRNAs in RPE1 cells. GL2 shRNA was a negative control. *A*, RPE1 cells transiently expressing GFP-tagged GPCRs were fixed and processed for immunofluorescence. Cilia were identified by staining endogenous ARL13B or acetylated tubulin. In each image, the cilium of interest is enlarged and highlighted in a box at the upper right corner. The scale bar represents 10 μm. *B*, bar graph showing the *CPIR*s of GFP-tagged GPCRs in (*A*). Data were from *n* = 3 independent experiments, with each experiment having ≥ 21 cells; error bars represent mean ± *SD*. *p* values were calculated using paired two-tailed *t*-tests. NS, not significant; ∗, *p* ≤ 0.05; ∗∗, *p* ≤ 0.005. GPCR, G protein–coupled receptor; *CPIR*, cilium-to-plasma membrane intensity ratio.

We investigated the role of Rab8-TNPO1 in the ciliary localization of GPCRs using lentivirus-transduced shRNA knockdown. Lentivirus-transduced shRNA targeting luciferase (GL2, negative control), Rab8, or TNPO1 was employed to perform knockdown in RPE1 cells. Subsequently, these cells were transfected with GFP-tagged GPCR constructs, including ADRB2, DRD1, DRD2, GPR161, GPR88, HTR6, NMUR1, NPY2R, and SMO (Fig. 3*A*). Compared to the control GL2 shRNA, Rab8 and TNPO1 shRNAs substantially reduced the endogenous level of target proteins in Western blot (Fig. S1, *A* and *B*). We also evaluated whether Rab8 or TNPO1 knockdown affected the GPCR expression levels. The total cellular GFP fluorescence was measured as a proxy for expression level, and we found no significant differences in GPCR expression across the knockdown conditions (*p* > 0.05, unpaired two-tailed *t*-tests) (Fig. S2).

Next, the ciliary localization of GPCRs was quantitatively assessed using the *CPIR* (Fig. 3*B* and Fig. S3, *A*–*I*). We performed three independent experiments. Individual datasets show a clear and consistent trend: of the nine GPCRs tested, knockdown of either Rab8 or TNPO1 clearly reduced the *CPIR*s for ADRB2, DRD2, GPR88, NMUR1, and NPY2R but not for DRD1, GPR161, or HTR6 in individual experiments (Fig. S3, *A*–*I*), consistent with our pull-down data. Since each independent experiment had matched conditions in control and knockdown, we performed paired two-tailed *t* test when pooling the three sets of data (Fig. 3*B*). The statistical analysis of the pooled data showed a similar trend to individual datasets, except for NMUR1, which did not show a significant change.

Interestingly, SMO did not interact with Rab8 and TNPO1 (Fig. 2, *F* and *I*), but Rab8 knockdown significantly reduced its ciliary localization (Figs. 3*B* and S3*I*). The observation suggests that Rab8 might indirectly regulate the ciliary localization of SMO, the underlying mechanism of which requires future investigation.

### Rab8-TNPO1 interacts with the C-terminal tail (CTT) of ADRB2

**Figure 4:**
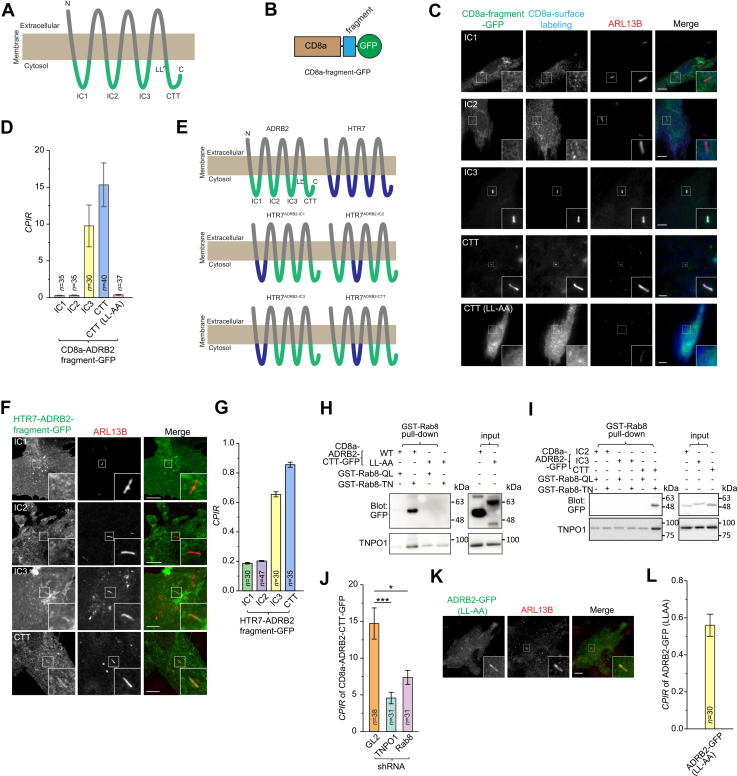
**Rab8 and TNPO1 interact with the CTT of ADRB2**. *A*, schematic diagram illustrating the domain organization of ADRB2. *B*, schematic diagram illustrating the domain organization of GFP-tagged CD8a-ADRB2 fragment chimeras. Fragments of ADRB2, including IC1-3 and CTT, were fused to the C terminus of cytosolic tail-deleted CD8a and the N terminus of GFP. *C*, surface labeling of CD8a was employed to reveal the ciliary localization of the CD8a-ADRB2-chimeras. RPE1 cells transiently expressing GFP-tagged CD8a-chimeras were surface-labeled for CD8a, fixed, and immunostained for endogenous ARL13B. The cilium is enlarged and highlighted in the box at the lower right corner of each image. The scale bar represents 10μm. *D*, bar graph showing the *CPIR*s of CD8a-ADRB2-chimeras imaged in (*B*). The number of cells used for analysis, *n*, is indicated. *E*, schematic diagram illustrating the domain organization of HTR7-ADRB2 chimeras, in which IC1-3 or the CTT of ADRB2 is replaced by that of HTR7. *F*, RPE1 cells transiently expressing indicated GFP-tagged HTR7-ADRB2 chimera were fixed and processed for immunofluorescence. Cilia were identified by immunostaining endogenous ARL13B. In each image, the cilium of interest is enlarged and highlighted in a box at the *bottom right corner*. The scale bar represents 10 μm. *G*, *CPIR* values of GFP-tagged HTR7-ADRB2 chimeras in (*F*). The number of cells used for analysis, *n*, is indicated; error bars represent mean ± *SEM*. *H*, GST-Rab8-TN specifically pulls down TNPO1 and WT CTT, but not the LL-AA mutant. Bead-immobilized GST-Rab8-QL or -TN was incubated with lysates from cells transiently expressing WT or LL-AA mutant CD8a-ADRB2-CTT-GFP. Retained proteins were analyzed by immunoblotting for GFP and endogenous TNPO1. *n* = 3 independent replicates were performed. *I*, GST-Rab8-TN specifically pulls down TNPO1 and CTT, but not IC2 and IC3. Bead-immobilized GST-Rab8-QL or -TN was incubated with lysates from cells transiently expressing GFP-tagged and CD8a-fused IC2, IC3, or CTT fragments. Retained proteins were analyzed by immunoblotting for GFP and TNPO1. *n* = 1 independent replicate was performed. Molecular weight markers (kDa) are labeled to the right of all immunoblots. *J*, bar graph showing the *CPIR*s of CD8a-ADRB2-CTT-GFP in RPE1 cells following knockdown of Rab8 and TNPO1. GL2 shRNA was a negative control. The ciliary localization was revealed by surface-labeling as described in (*C*). *n* indicates the number of cells analyzed; error bars represent mean ± *SEM*. *p* values are from unpaired two-tailed *t*-tests; ∗, *p* ≤ 0.05; ∗∗∗, *p* ≤ 0.0005. *K*, the LL-AA mutant ADRB2 localizes to the cilium. RPE1 cells transiently expressing ADRB2-GFP with LL-AA mutation were fixed and processed for immunofluorescence. Cilia were identified by staining endogenous ARL13B. The scale bar represents 10 μm. The cilium is enlarged and highlighted in the box at the *lower right corner* of each image. Its corresponding *CPIR* is shown in (*L*). *L*, bar graph showing the *CPIR* of ADRB2-GFP (LL-AA) imaged in (*K*). *n* indicates the number of cells analyzed; error bars represent mean ± *SEM*. The number of independent replicates in (*D*), (*G*), (*J*), and (*L*) is one. CTT, C terminus cytosolic tail; ADRB2, β2 adrenergic receptor; IC3, intracellular loop 3; *CPIR*, cilium-to-plasma membrane intensity ratio; HTR7, 5-hydroxytryptamine receptor 7; LL, dileucine motif in the CTT.

Our data suggest that Rab8-TNPO1 targets GPCRs to the cilium by interacting with their CTSs. The CTSs of the five Rab8-TNPO1-dependent GPCRs were not studied. We selected ADRB2 for a detailed investigation of its CTS. A GPCR contains four cytosolic regions: intracellular loops 1 to 3 (IC1-3) and the CTT (Fig. 4*A*). To investigate which of the four regions contains the CTS, we replaced the CTT of CD8a, a type I transmembrane protein with weak and nonspecific ciliary localization (15), with IC1-3 or CTT, followed by a GFP tag (Fig. 4*B*). Ciliary localization was assessed using surface staining of the extracellularly exposed CD8a ectodomain. Although CD8a-fused IC1 and IC2 were cilium negative, interestingly, either intracellular loop 3 (IC3) (CD8a-IC3-GFP) or CTT (CD8a-CTT-GFP) was sufficient to localize CD8a to the cilium (Fig. 4*C*), suggesting that ADRB2 might possess two CTSs. Quantitative analysis showed that the *CPIR*s of IC1 and IC2 were comparable to that of CD8a-GFP, which exhibits a nonspecific weak ciliary localization with a *CPIR* of 0.25 (15) (Fig. 4*D*). In contrast, the *CPIR*s of IC3 and CTT were more than 20-fold higher than those of IC1 and IC2 (Fig. 4*D*), supporting that these regions containing a CTS.

One caveat is that intracellular loops are normally membrane-anchored at both ends, whereas when fused to the C terminus of CD8a, only their N-termini remain membrane-anchored, potentially preventing them from adopting their native conformations. To exclude the possibility that the CTS resides within IC1 or IC2, we generated HTR7 chimeras in which IC1–3 or the CTT was replaced with the corresponding region of ADRB2 (Fig. 4*E*). Consistent with the CD8a chimeras, HTR7 chimeras containing ADRB2 IC3 or CTT displayed robust ciliary localization, whereas those containing ADRB2 IC1 or IC2 showed weak or no ciliary localization (Fig. 4*F*). Supporting this observation, the *CPIR* values of HTR7 chimeras with ADRB2 IC1 or IC2 were comparable to Flag-HTR7, whereas those containing ADRB2 IC3 or CTT were comparable to ADRB2-GFP (Fig. 4*G*). Together, these data demonstrate that ADRB2 IC3 and the CTT function as the CTS.

To explore the underlying mechanism, we performed pull-down assays. Bead-immobilized GST-Rab8-TN, but not GST-Rab8-QL, specifically interacted with the CTT (CD8a-CTT-GFP) but not with IC3 (CD8a-IC3-GFP) (Fig. 4, *H* and *I*). Consistently, TNPO1 was pulled down only when CD8a-CTT-GFP was retained by GST-Rab8-TN, indicating the cooperative involvement of Rab8-GDP in binding to TNPO1 and CTT. This finding aligns with a previous report by. (35), which demonstrated that the CTT of ADRB2 preferentially interacts with Rab8 in its GDP-bound form. Our study extends this observation by uncovering the involvement of TNPO1 in this interaction.

Dong *et al*. also identified the double leucine motif (LL) at the juxtamembrane region of the CTT as essential for its interaction with Rab8 (35). To assess the role of this motif, we mutated the double leucine to alanine (LL-AA) in CD8a-CTT-GFP. This mutation abolished its ciliary localization (Fig. 4, *C* and *D*) and disrupted its interaction with GST-Rab8-TN in pull-down assays (Fig. 4*H*). Consistent with the importance of the LL-motif in CTT-mediated ciliary targeting, knocking down Rab8 or TNPO1 significantly reduced the *CPIR* of CD8a-CTT-GFP (Fig. 4*J*).

Interestingly, full-length ADRB2 with the LL-AA mutation retained ciliary localization (Fig. 4*K*), with a *CPIR* (0.56 ± 0.06) (Fig. 4*L*). This observation suggests that IC3 might partially compensate for the loss of function of the CTT in ciliary targeting. Therefore, our findings indicate that ADRB2 might contain two CTSs, IC3 and CTT. Since the knockdown of Rab8 or TNPO1 significantly reduced the *CPIR*s of ADRB2 (Fig. 3*B*), we propose that the two CTSs might additively contribute to the ciliary localization of ADRB2. Although Rab8-TNPO1 serves as the transport adapter for the CTT, the adapter for IC3 remains unidentified.

## Discussion

We screened nine cilium-localized GPCRs to assess their interaction with Rab8-TNPO1 and their dependence on it for ciliary localization. Together with our previous work (15), we identified six ciliary GPCRs—ADRB2, DRD2, GPR88, NMUR1, NPY2R, and RHO—that interact with Rab8-TNPO1. Consistent with our biochemical interaction data, the knockdown of Rab8 or TNPO1 compromised the ciliary localization of these Rab8-TNPO1-interacting GPCRs, therefore establishing these GPCRs as potential clients of the ciliary transport adapter, Rab8-TNPO1. Since GPCR conformation and activity can be sensitive to detergent choice, our interaction assays may be limited by the use of Triton X-100 during lysate preparation. It is possible that certain interactions could be disrupted under these conditions. Future studies will benefit from screening and optimizing milder detergents to better preserve GPCR structure and function.

Compared to similar studies, our findings provide robust biochemical evidence for specific interactions between GPCRs and Rab8-TNPO1, using the differential interaction profiles of Rab8-TN and Rab8-QL as stringent controls. These results are summarized in Table 1, which also includes comparative data from studies by the Jackson and Mukhopadhyay labs on the ciliary localization of GPCRs following TULP3 depletion or KO (12, 14, 30). The data suggests that Rab8-TNPO1 and TULP3 have distinct as well as overlapping client sets. Investigating how these two ciliary transport adapters coordinate to transport shared clients into the cilium will present an intriguing avenue for future research.

Except for GPR161, RHO, and SMO, the GPCRs examined in this study are expected to function in the neuronal cilium under native conditions (3). However, a limitation of our study, as with many similar studies, is the use of nonneuronal RPE1 and HEK293T cells. These cell lines may lack essential ciliary trafficking factors, potentially misleading our conclusions about ciliary targeting.

Previously, we identified ARL13B, PKHD1, RDH8, RHO and RP2 as Rab8-TNPO1 clients for ciliary transport (15, 16). Our current study significantly expands the list of Rab8-TNPO1 clients, by establishing Rab8-TNPO1 as a ciliary transport adapter for five GPCRs. It is likely that many additional GPCRs also rely on Rab8-TNPO1 for their ciliary localization.

## Experimental procedures

### DNA plasmids

Table S1 for a list of plasmids used in this study.

### Antibodies

Mouse monoclonal antibodies against acetylated α-tubulin (Merck, #T6793, 1:1000 for immunofluorescence or IF), TNPO1 (Abcam, #ab10303, 1:3000 for Western blotting or WB), Rab8 (BD biosciences, #610844, 1:1000 for WB), GFP (Santa Cruz, #sc9996, 1:1000 for WB), and CD8a (Developmental Studies Hybridoma Bank, clone OKT8, 1:200 for IF). Rabbit polyclonal antibodies against ARL13B (15); 1:1000 for IF). Horseradish eroxidase–conjugated goat anti-mouse and anti-rabbit IgG antibodies were purchased from Bio-Rad. Alexa Fluor-conjugated goat anti-mouse and anti-rabbit IgG antibodies (1:500 for IF) were purchased from Invitrogen.

### Cell culture and transfection

RPE1 and HEK293T cells were from the American Type Culture Collection, and HEK293FT cells were from Thermo Fisher Scientific. RPE1 cells were cultured in Dulbecco’s Modified Eagle’s Medium (DMEM) and Ham’s F12 mixture medium supplemented with 10% fetal bovine serum (FBS). HEK293T cells were cultured in DMEM supplemented with 10% FBS. HEK293FT cells were cultured in DMEM supplemented with 10% FBS and 500 μg/ml G418 (Thermo Fisher Scientific). Transfection of plasmid DNA was performed using Lipofectamine 2000 (Thermo Fisher Scientific), according to the manufacturer’s protocol. Ciliogenesis was induced by incubating cells in the serum-free medium for 48 h.

### CPIR calculation

*CPIR* calculation was performed as described previously (15). Briefly, a line intensity profile was generated by drawing a line approximately 1 μm wide orthogonally across the cilium, from which the maximum intensity value (*I*_max_) was obtained. The mean intensity of PM (*I*_PM_) and the background intensity value (*I*_background_) were determined by placing circular regions of interest over the PM and a noncellular background area, respectively. The *CPIR* of the membrane protein is defined as (*I*_max_ − *I*_PM_)/(*I*_PM_ − *I*_background_). This metric reflects the relative enrichment of a membrane protein in the cilium, normalized to the PM signal (for CD8a chimeras) or the general cellular pool (for GFP-tagged GPCRs).

### Immunofluorescence staining and microscopy

RPE1 cells were seeded on No. 1.5 12/25-mm coverslips in a 24/6-well plate, respectively. Cells were serum-starved for 48 h to induce ciliogenesis, then fixed with 4% paraformaldehyde in PBS and subsequently neutralized with ammonium chloride. The cells were incubated with primary antibodies diluted in antibody dilution buffer (PBS supplemented with 5% FBS and 2% bovine serum albumin and 0.1% Saponin (Sigma-Aldrich)). After several washes, cells were incubated with secondary antibodies diluted in antibody dilution buffer. The coverslips were mounted in Mowiol 4 to 88 (EMD Millipore) after extensive washes using PBS.

To surface label the CD8a-tagged chimeras, cells were transfected to express CD8a-fused cytosolic fragments of ADRB2. The selective labeling of the surface-exposed pool of chimera was performed by first incubating with anti-CD8a antibody on ice for 1 h. After washing away unbound antibody, cells were processed for immunofluorescence staining. Cells were imaged under an inverted wide-field Olympus IX83 microscope system equipped with a Plan Apo oil objective lens (63 × or 100 × , NA 1.40), a motorized stage, a focus drift correction device, motorized filter cubes, a scientific complementary metal oxide semiconductor camera (Neo; Andor Technology), and a 200 W metal halide excitation light source (Lumen Pro 200; Prior Scientific). Dichroic mirrors and filters in filter turrets were optimized for GFP/Alexa Fluor 488, mCherry/Alexa Fluor 594, and Alexa Fluor 647. The microscope system was controlled by MetaMorph software (Molecular Devices).

### Knockdown of Rab8 and TNPO1

Endogenous Rab8 and TNPO1 were depleted by lentivirus-mediated transduction of shRNA. DNA plasmids, including GL2 shRNA, TNPO1 shRNA or Rab8 shRNA in pLKO.1 vector, together with the packaging plasmids pLP1, pLP2, and pLP/VSVG (Thermo Fisher Scientific), were transiently transfected to HEK293FT cells using Lipofectamine 2000. Lentiviruses were harvested at 36 and 60 h posttransfection, filtered through a 0.45 μm filter (Sartorius), and immediately used to infect RPE1 cells. RPE1 cells seeded in a 6-well plate were infected twice every 24 h with filtered lentiviruses using 8 μg/ml hexadimethrine bromide (Sigma-Aldrich #H9268). The cells were subsequently subjected to transient transfection to express a GFP-tagged GPCR. They were then split into a 24-well plate for Western blot analysis of knockdown efficiency or onto coverslips followed by serum starvation for 48 h to induce ciliogenesis before immunolabeling.

### Purification of his- and GST-tagged fusion proteins

pET30ax DNA plasmids encoding His-tagged Rab8-TN and Rab8-QL were transformed into BL21 *E*. *coli* cells. Transformed bacteria were induced by IPTG, subsequently pelleted and lysed by sonication in lysis buffer (50mM Tris, pH 8.0, 100 mM NaCl, 0.1% Triton X-100 and 1mM DTT). The lysate was cleared by centrifugation, and the supernatant was incubated with pre-washed Ni-NTA agarose beads (QIAGEN) in the presence of 10 mM imidazole in a cold room overnight. Beads were washed with the lysis buffer containing 25 mM imidazole and the bound protein was eluted with the lysis buffer containing 250 mM imidazole. The eluted protein was dialyzed and concentrated by ultrafiltration spin column (GE Healthcare), quantified by Coomassie gel staining, and stored in −20 °C supplemented with 50% glycerol until use. pGEB and pGEX-4T1 DNA plasmids encoding GST-tagged TNPO1, Rab-TN, and Rab-QL were transformed into BL21*E*. *coli* cells, induced by IPTG, subsequently pelleted and lysed by sonication in lysis buffer (50mM Tris, pH 8.0, 100 mM NaCl, 0.1% Triton X-100, and 1mM DTT). The lysate was cleared by centrifugation and the supernatant was incubated with pre-washed glutathione Sepharose (GE Healthcare) in a cold room overnight. Beads were washed by the lysis buffer and the bound protein was quantified by Coomassie gel staining and stored in −20 °C supplemented with 50% glycerol until use.

### Pull-down experiments

HEK293T cells were used for all the interaction assays to express various tagged proteins. After 24 to 36 h of transfection as mentioned above, cells were lysed in the lysis buffer containing 40 mM hepes pH 7.3100 mM NaCl, 1% Triton X-100 and 1mM DTT. The resulting lysates were incubated in a cold room for 30 min, centrifuged at 17,000*g* for 30 min, and the supernatant was incubated with 10 to 40 μg of bead-immobilized GST-fusion protein overnight. For the GST-TNPO1 pull-down, 10 μg of His-tagged Rab8-TN or Rab8-QL was also added to the lysate. The beads were washed with the lysis buffer, and the bound proteins were eluted by boiling them in the SDS sample buffer and resolved by SDS-PAGE. The separated proteins were transferred to a polyvinyl difluoride membrane (Bio-Rad) and subsequently incubated with primary and horseradish peroxidase–conjugated secondary antibodies. Western blot and molecular weight marker bands were visualized using chemiluminescence and white light illumination, respectively, with a cooled charge-coupled device (LAS-4000, GE Healthcare Life Sciences). Molecular weights were assigned by manually aligning the two images. Uncropped blot images are presented in Figure S4.

### Image and statistical analysis

All image analysis was conducted using ImageJ software (https://imagej.nih.gov/ij/). Data analysis and graphs were plotted using OriginPro 9 (OriginLab). All images were randomly taken and included in analyses. The sample size, *n*, is indicated wherever applicable in figures or corresponding legends. A two-tailed unpaired *t* test analysis was conducted in Excel (Microsoft) to assess statistical significance. A *p* value of less than 0.05 was considered statistically significant.

## Data availability

All data are included in the article and supplementary information.

## Supporting information

This article contains supporting information (36).

## Conflict of interest

The authors declare that they have no conflicts of interest with the contents of this article.

## Supporting information

Fig S1–S4

TableS1
