# Supplementary figures and images for: Rab8 and TNPO1 function as the ciliary transport adapters for GPCRs
Source: J Biol Chem. 2026 Apr 24;302(6):112202. doi: 10.1016/j.jbc.2026.112202 (PMC13202561; doi:10.1016/j.jbc.2026.112202)

# Supplementary Figure 1

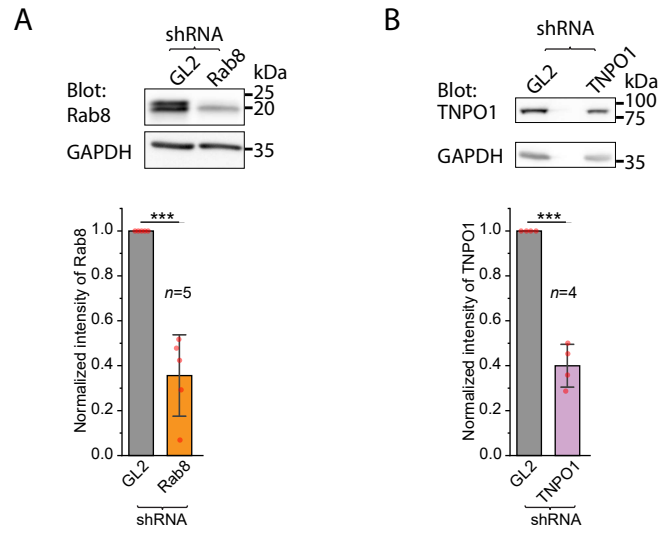

Supplementary Figure 2

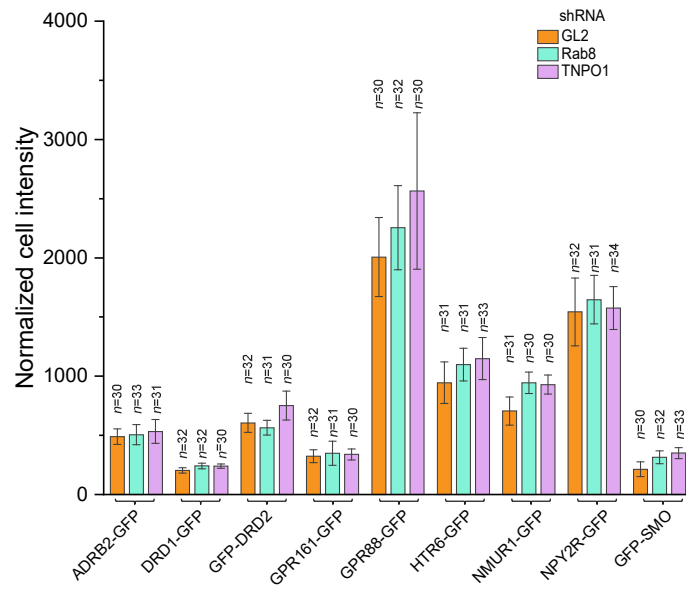

Supplementary Figure 3

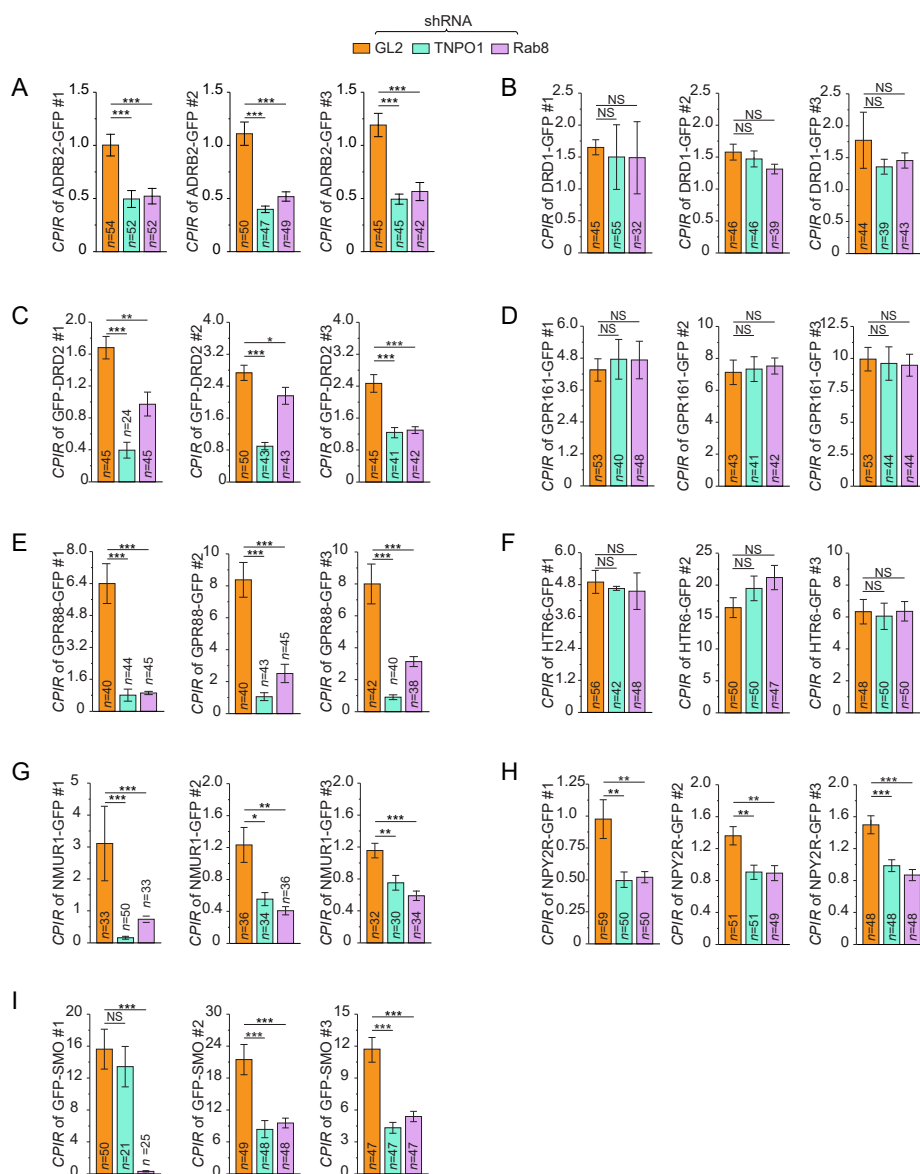

# Supplementary Figure 4

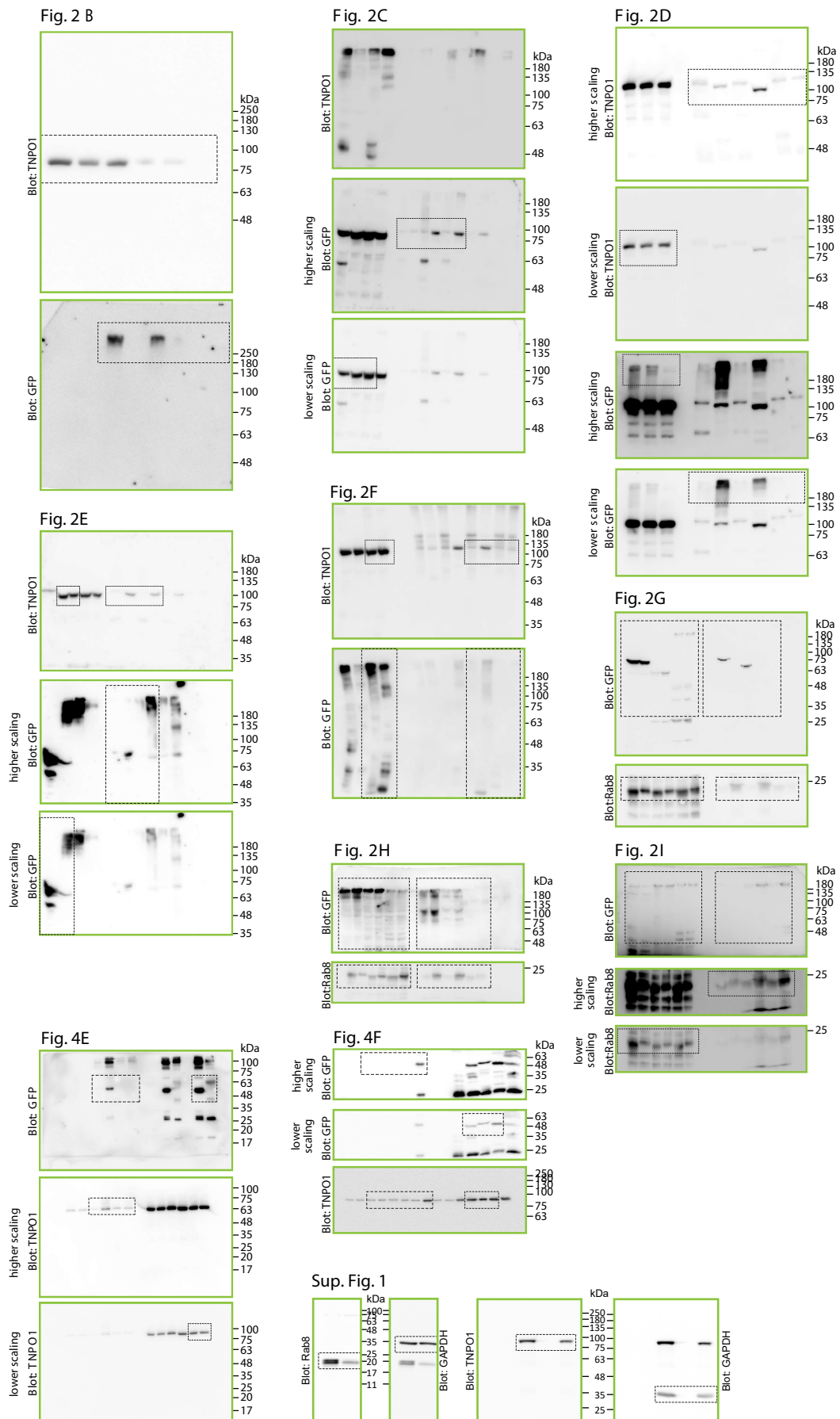

Supplement: Fig S1–S4 [file mmc1.pdf]
