# Supplementary material for: Rab8 and TNPO1 function as the ciliary transport adapters for GPCRs
Source: J Biol Chem. 2026 Apr 24;302(6):112202. doi: 10.1016/j.jbc.2026.112202 (PMC13202561; doi:10.1016/j.jbc.2026.112202)
Supplement: Supplementary material [file mmc3.docx]

**Rab8 and TNPO1 function as the ciliary transport adaptors for GPCRs**

Divyanshu Mahajan^1^, Viswanadh Madugula^1^, Hui Min Chia^1^, and Lei Lu^1*^

**Supporting Information**

**Supplementary table**

**Supplementary Table 1**

DNA plasmids used in this study.

**Supplementary figure legend**

**Supplementary Figure 1**

After lentivirus-mediated transduction of Rab8, TNPO1, or GL2 shRNA, RPE1 cell lysates were subjected to immunoblotting for endogenous Rab8, TNPO1, or GAPDH (loading control). The bar graph below shows GAPDH-normalized band intensities of Rab8 (**A**) and TNPO1 (**B**). *n* indicates the number of independent experiments. Molecular weight markers (kDa) are labeled to the right of all immunoblots.

**Supplementary Figure 2**

Cellular expression levels of GPCRs are not affected by Rab8 or TNPO1 knockdown. RPE1 cells were subjected to knockdown and GFP-tagged GPCR expression as described in Figure 3. Total GFP fluorescence intensities of individual cells were quantified and plotted. *n* indicates the number of cells analyzed; error bars represent mean ± *SEM*.

**Supplementary Figure 3**

(**A-I**) Three individual datasets showing the *CPIR*s of GFP-tagged GPCRs upon the knockdown of GL2, Rab8, or TNPO1. *n* indicates the number of cells analyzed; error bars represent mean ± *SEM*. *p* values were calculated using unpaired two-tailed *t*-tests. NS, not significant; *, *p* ≤ 0.05; **, *p* ≤ 0.005; ***, *p* ≤ 0.0005.

**Supplementary Figure 4**

Uncropped gel blot images used to prepare Figure 2, Figure 4 and Supplementary Figure 1. Green box, chemiluminescence image; black box, cropped region shown in the corresponding figures. Molecular weight markers (kDa) are labeled to the right of all immunoblots.
